# The role of immunotherapy in solid tumors: report from the Campania Society of Oncology Immunotherapy (SCITO) meeting, Naples 2014

**DOI:** 10.1186/s12967-014-0291-1

**Published:** 2014-10-21

**Authors:** Paolo A Ascierto, Raffaele Addeo, Giacomo Cartenì, Bruno Daniele, Michele De Laurentis, Giovanni Pietro Ianniello, Alessandro Morabito, Giovannella Palmieri, Stefano Pepe, Francesco Perrone, Sandro Pignata, Vincenzo Montesarchio

**Affiliations:** Unit of Melanoma, Cancer Immunotherapy and Innovative Therapies, Istituto Nazionale Tumori Fondazione “G. Pascale”, Via Mariano Semmola, 80131 Naples, Italy; Unit of Oncology, Ospedale “San Giovanni di Dio”, Frattamaggiore, NA Italy; Unit of Medical Oncology, Dipartimento di Oncopneumoematologia, A.O.R.N. “A. Cardarelli”, Naples, Italy; Department of Oncology, A.O. “G. Rummo”, Benevento, Italy; Unità Oncologia Medica Senologica, Istituto Nazionale Tumori Fondazione “G. Pascale”, Naples, Italy; Department of Oncology, A.O.R.N. “S. Anna e S. Sebastiano”, Caserta, Italy; Unità Oncologia Medica Toraco-Polmonare, Istituto Nazionale Tumori Fondazione “G. Pascale”, Naples, Italy; Department of Molecular and Clinical Endocrinology and Oncology, University “Federico II”, Naples, Italy; Dipartimento di Medicina e Chirurgia, A.O.U. “San Giovanni di Dio e Ruggi d’Aragona”, University of Salerno, Salerno, Italy; Unità Sperimentazioni Cliniche, Istituto Nazionale Tumori Fondazione “G. Pascale”, Napoli, Italy; Dipartimento di Oncologia Uroginecologica, Istituto Nazionale Tumori Fondazione “G. Pascale”, Naples, Italy; Unit of Oncology, A.O.R.N. dei COLLI “Ospedali Monaldi-Cotugno-CTO”, Naples, Italy

**Keywords:** Immunotherapy, Checkpoint inhibitors, Cellular vaccine, Antigen-specific vaccines, Solid tumors

## Abstract

The therapeutic approach to advanced or metastatic solid tumors, either with chemotherapy or targeted therapies, is mainly palliative. Resistance to chemotherapy occurs very frequently and is one of the most important reasons for disease progression. Immunotherapy has the potential to mount an ongoing, dynamic immune response that can kill tumor cells for an extended time after the conventional therapy has been administered. Such a long-lasting response is potentially able to completely eradicate tumor cells, rather than producing only a temporary killing of cells. The most promising immune-based treatments are monoclonal antibodies that act as checkpoint inhibitors (e.g. ipilimumab and nivolumab), adoptive cell therapy (e.g. T-cells expressing chimeric antigen receptors) and vaccines (e.g. sipuleucel-T). Ipilimumab is currently approved for the treatment of metastatic melanoma and sipuleucel-T is approved for advanced prostate cancer. There is great interest in immunotherapy in other solid tumors, potentially used alone or in a multimodal fashion with chemotherapy and/or biological drugs. In this paper, we review recent advances in immuno-oncology in solid malignancies (except melanoma) as were discussed at the inaugural meeting of the Campania Society of Oncology Immunotherapy (SCITO).

## Introduction

The immune system is able to recognize and eradicate cancer cells via multiple and complex mechanisms. Ehrlich first proposed, in 1909, the idea that the immune system could search and attack transformed cells before they are clinically visible. Years later, this was confirmed by studies involving tumor transplantation models that suggested the existence of tumor-associated antigens and formed the basis of immune surveillance [1].

The immune system can be divided into innate and adaptive. Innate immunity commonly refers to myeloid and lymphoid cells that exert a rapid effector function, while adaptive immunity is driven by T- and B-lymphocytes that express antigen receptors produced by site-specific somatic recombination. Adoptive immunity has greater specificity than innate in retaining antigen memory. The broadness and quality of a T-cell response is regulated by a balance of activating and inhibitory signals. In this scenario, checkpoints are placed to limit an ongoing immune response, thereby preventing damage to healthy tissues. PD-1, CTLA-4, and LAG-3 are examples of inhibitory checkpoints.

In human cancer, the immune system plays a double role, both protecting against tumor development and promoting tumor growth. This process is known as immunoediting and has three well-defined phases [2]. The immunosurveillance (elimination) phase is characterized by antigen presentation and T cell activation and, more importantly, by destruction of nascent tumor cells and control of tumor growth. In the equilibrium phase, the main features are genetic instability and tumor heterogeneity, leading to a steady-state between tumor growth enhancement and inhibition. In the escape phase, cancer progression is favoured by the outgrowth of tumor cells that can suppress or escape the immune system. T-regulatory (T-reg) cells are crucially involved at this stage. Tumor-infiltrating lymphocytes (TILs) have been identified in many tumor types and often have prognostic value. The presence of intratumoral T-cells strongly correlates with improved clinical outcome in advanced ovarian carcinoma [3] and in other solid tumors including non-small cell lung (NSCLC) [4], colorectal [5], breast [6], head and neck [7] and kidney cancer [8] as well as melanoma [9]. Conversely, T-reg infiltration has been reported to predict a poorer outcome in early-stage NSCLC [10], in melanoma [11], and in renal cell carcinoma [12].

### Checkpoint blockade: now a reality?

The two main inhibitory checkpoint pathways involve signaling through CTLA-4 or PD-1. Both systems are crucial in promoting tumor growth and proliferation: CTLA-4 is competitive for the costimulatory binding CD80/86-CD28 and its binding to CD80/86 generates a negative signal which is responsible for immune cell inactivation. PD-1 binding to PD-L1 and PD-L2 molecules also generates a negative and inhibitory signal responsible for immune escape. The CTLA-4 pathway is more important in the early phase of the immune system activation (priming phase), while the PD-1 pathway is more important in the tumor microenvironment during the effector phase [13,14]. Inhibition of CTLA-4 and PD-1 binding to their ligands enhances T-cell activation and proliferation, leading to tumor infiltration by T-cells and tumor regression.

The anti-CTLA-4 monoclonal antibody (moAb) ipilimumab was the first therapy to improve overall survival (OS) in a phase III trial in patients with metastatic melanoma, when compared with GP100, a peptide vaccine [15]. Progression-free survival (PFS) and best overall response rate (BORR) also favored patients receiving ipilimumab, alone or in combination with GP100, as compared with the vaccine alone. Most adverse events (AEs) reported with ipilimumab were immune-related (irAEs) and were managed with specific algorithms [16]. The most frequently reported irAEs in the ipilimumab arm were diarrhea (28%), pruritus (24%) and rash (19%).

When the PD-1 receptor binds with its ligand (PD-L1/B7-H1), which is frequently overexpressed on tumor cell surfaces, T-cell inhibition and down-regulation of T-cell responses occurs. This allows tumors to directly halt antitumor T-cell activity, also known as adaptive resistance. Blocking PD-1 or PD-L1 through the use of therapeutic moAbs empowers the T-cell response. Promising long-term survival results have been achieved with the anti-PD1 moAb nivolumab. In a phase I trial in patients with advanced solid tumors, nivolumab was associated with a 2-year survival rate of 24% in NSCLC, 43% in melanoma, and 50% in renal cell carcinoma [17-19]. Patients treated with 3 mg/kg or 10 mg/kg had an objective response rate (ORR) greater than those treated with 1 mg/kg (24% and 20% versus 3%). Grade 3–4 AEs were reported in 17% of patients, mainly rash, diarrhea and pruritus. The nivolumab 3 mg/kg dose reached a median OS of 14.9 months and was selected for future registration trials.

Immune checkpoints inhibitors have different toxicity profiles compared to chemotherapy or targeted therapies (Table 1). Some AEs have different etiologies compared with those related to chemotherapy (e.g. diarrhea, rash) and require different management. These irAEs result from increased activity of the immune system, can involve multiple organs and may be severe or life-threatening. In such cases, systemic high-dose corticosteroids may be required. Patient education is essential in order to early recognize irAEs and to minimize life-threatening complications.

**Table 1:** **Summary of selected adverse events reported with immune checkpoints inhibitors**

**Category**	**Adverse events**
Common	
Skin	Pruritus, rash, vitiligo, urticaria, alopecia, macular rash, hypopigmentation, erytema, erytematous rash
Gastrointestinal	Diarrhea, colitis, nausea, abdominal pain
Endocrine	Hypothyroidism, hyperthyroidism, hypopituitarism, hypophysitis, adrenal insufficiency, altered hormone levels
Hepatic	Hepatitis, increased liver function enzymes
Pulmonary	Pneumonitis, pulmonary edema
Uncommon	
Ocular	Uveitis, episcleritis, eye pruritus
Pancreatic	Elevated lipase levels, hyperglycemia
Infusion-related	Infusion-related reactions, hypersensitivity reactions
Hematologic	Anemia, leukocytosis, thrombocytopenia
Neurologic	Peripheral neuropathies, headache
General	Fatigue, decreased appetite, arthralgia

Predictive biomarkers for immuno-oncology therapies, to be correlated with efficacy and toxicity, are under investigation. However, due to their being directed against the patient’s immune system rather than the tumor, a different approach for identifying biomarkers in this field may be needed [20]. A promising approach could be the expression of biomarkers related to the target pathway. For example, PD-L1 seems an effective biomarker for therapies directed against the PD-1 pathway, such as nivolumab and pembrolizumab. Some studies in NSCLC and melanoma showed that the ORR in patients with PD-L1 positivity were higher when compared withPD-L1 negative patients (Table 2) [17,21,22]. However, key questions remain over variability in tissue collection time, cell sampling, type of assay and immunohistochemical (IHC) criteria to determine a significant cut-off between wild-type and mutation status.

**Table 2:** **PD-L1 as a potential efficacy biomarker: response according to PD-L1 expression in NSCLC and melanoma**

	**Tumor**	**PDL-1 + ve**	**PDL-1-ve**
		**ORR n/N (%)**	**ORR n/N (%)**
MPDL3280A Hamid et al. ASCO #9010	Melanoma	4/15 (27%)	3/15 (20%)
Nivolumab Weber et al. ASCO #9011	Melanoma	8/12 (67%)	6/32 (19%)
Nivolumab Grosso et al. ASCO #3016	Melanoma	7/16 (44%)	3/18 (17%)
Nivolumab Topalian et al. NEJM 2012	Melanoma	9/25 (36%)	0/17 (0%)
Nivolumab Antonia et al. WCLC 2013	NSCLC	5/31 (16%)	4/32 (13%)
Pembrolizumab Garon et al. WCLC 2013	NSCLC	4/7 (57%)	2/22 (9%)
MPDL3280A Horn et al. WCLC2013	NSCLC	8/26 (31%)	4/20 (20%)
Nivolumab/ipilimumab Callahan et al. ASCO#3003	Melanoma	4/10 (40%)	8/17 (47%)

In order to maximise clinical benefit, multiple sequential or combination approaches between immunotherapy, chemotherapy, radiotherapy and targeted drugs are under investigation. Clinical trials combining immuno-oncology therapies that modulate different pathways or target distinct and potentially complementary immune pathways (e.g. anti-PD-1 plus anti-CTLA-4 or anti-LAG3 antibodies) are ongoing in advanced solid tumors. The synergistic activity between immuno-oncology therapies and chemotherapy has also been extensively demonstrated both in vitro and in vivo. For example, ipilimumab in combination with dacarbazine (DTIC) showed a long-term survival benefit compared with placebo plus DTIC in patients with metastatic melanoma [23].

### The need for new endpoints in immuno-oncology

The value and the meaning of endpoints in the development of new anticancer drugs has been a topic of keen interest in recent years. Targeted drugs can interfere with tumor cell growth in a substantially different manner to cytotoxic drugs. Thus, the need to observe tumor shrinkage in order to consider a drug active has been questioned. Indeed, with such drugs, it was anticipated that slowing the growth of tumor cells might be clinically meaningful even in the absence of significant tumor shrinkage. Drugs interfering with the immune system pose other different problems, in that stimulation of immunocompetent cells may produce an infiltration of tumor masses that tend to increase the volume of the lesion, thereby counterbalancing the reduction in tumor cells. Such phenomenon translates into the need to review objective response (OR) criteria, because common RECIST and WHO criteria might not be adequately sensitive.

Phase I studies in melanoma clearly illustrate that an initial increase in tumor volume or the appearance of new lesions does not necessarily mean therapeutic failure [24] and that stable disease (SD) may be an indicator of a clinically meaningful therapeutic effect. In a nivolumab phase I trial, it was shown that ORs may occur early or late and may also continue after drug discontinuation [17]. Among responders who stopped treatment for reasons other than progressive disease (PD) (n = 27), 70% maintained the response off-drug for 16 to 59 weeks.

**Figure 1:**
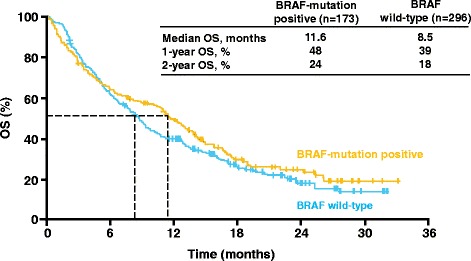
**Ipilimumab clinical activity (OS) irrespective of BRAF mutational status: the Italian EAP in melanoma by Ascierto et al. J Trans Med 2014; 12: 116–122 [** 26 **].**

**Figure 2:**
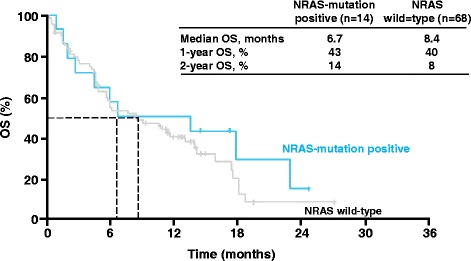
**Ipilimumab clinical activity (OS) irrespective of NRAS mutational status: the Italian EAP in melanoma by Ascierto et al. J Trans Med 2014; 12: 116–122 [** 26 **].**

Anti-CTLA-4 and anti-PD1 moAbs may act regardless of patient characteristics (age, gender, ECOG PS) and disease characteristics (histology, mutation status, type of prior therapies). This was observed with nivolumab in NSCLC [25] and ipilimumab in melanoma [26]. In the Italian extended-access programme (EAP), ipilimumab was shown to be active irrespective of mutational status: disease control rates (DCR) and OS were comparable between BRAF or NRAS-mutated and wild-type patients in a very large population [27] (Figures 1 and 2). Across the phase II-III clinical trial program, four patterns of response to ipilimumab in patients with advanced melanoma were observed and named as immuno-related response criteria (irRC): (1) response in baseline lesions; (2) SD with slow, steady decline in total tumor volume; (3) response after initial increase in total tumor volume; (4) reduction in total tumor burden after the appearance of new lesions [28]. In the CA184-008 and CA184-022 trials of ipilimumab in metastatic melanoma, the tumor responses of 167 evaluable patients have been assessed with the irRC [29,30]. Twenty-two patients were characterized as having an immuno-related partial response (PR) (n = 5) or irSD (n = 17), who otherwise would be labelled as PD by conventional WHO criteria.

Such considerations imply that tumor assessments should be performed only after completion of the assigned regimen and the results confirmed with a follow-up scan [28]. With drugs such as ipilimumab, the planned treatment should be administered regardless of the early appearance of new lesions or volume increase of existing lesions, as immune cell infiltration following immunotherapy may mimic tumor progression. Data from clinical trials and ipilimumab EAPs indicate that long-lasting SD is a common outcome with immunotherapy and that, even with no evidence of a tumor response, durable disease control can result in prolonged OS [31]. Such evidence clearly suggests that classical response rates (RR) and PFS cannot be considered as valid surrogate of OS or long-term clinical benefit. Before definitive trials that address OS as end-point are available, other surrogate indicators (e.g. rate of survival at a predefined time point) are becoming more relevant since they may be more predictive of survival duration [32].

### Immunotherapy of solid tumors

#### Lung cancer

Lung cancer has historically been considered as a non-immunogenic cancer, so immunotherapy has not been extensively studied in this field. However, more recently, advances in the understanding of antitumor immune evasion and response mechanisms have led to preliminary positive results with different immuno-oncology strategies in NSCLC. The first evidence of efficacy with a moAb in combination with chemotherapy in advanced NSCLC has been obtained with ipilimumab in a randomized phase II trial [33]. Treatment-naïve patients with stage IIIB/IV NSCLC were randomized to receive standard chemotherapy (carboplatin plus paclitaxel q3w) in combination with placebo or ipilimumab in one of the following regimens: concurrent (four doses of ipilimumab plus paclitaxel and carboplatin followed by two doses of placebo plus paclitaxel and carboplatin) or phased (two doses of placebo plus paclitaxel and carboplatin followed by four doses of ipilimumab plus paclitaxel and carboplatin). Maintenance therapy with placebo or ipilimumab was administered for 12 weeks following the induction phase. The study met its primary endpoint of improved irPFS versus the control arm but only for phased ipilimumab (HR 0.72; p = 0.05) and not for the concurrent arm (HR 0.81). Improvement in irPFS was greater in patients with squamous histology (HR 0.55). Another randomized phase II trial, with the same design and treatment arms, enrolled patients with advanced small-cell lung cancer (SCLC), confirming the benefit in irPFS of ipilimumab plus chemotherapy versus chemotherapy alone [34]. However, both trials failed to show any significant OS difference between the arms, probably due to being underpowered for this endpoint. Currently, there are two ongoing multicenter, randomized, placebo-controlled phase III trials aiming to detect an OS benefit by adding ipilimumab to standard chemotherapy in patients with SCLC and squamous NSCLC. Tremelimumab, another anti-CTLA4 moAb, has also been studied in the maintenance setting for NSCLC patients with stable or responding disease after first-line chemotherapy, showing no improvement in PFS as compared with best supportive care (BSC) [35].

PD-1 inhibitors, such as nivolumab or pembrolizumab, are potentially active in NSCLC because PD-1 receptors are highly expressed on NSCLC infiltrating T-cells. CA209-003/CHECKMATE-003 is a phase I trial assessing the safety, antitumor activity, pharmacodynamics and pharmacokinetics of nivolumab in patients with advanced solid tumors, including NSCLC [18]. The NSCLC cohort (n = 129) has been randomised to receive three nivolumab dose levels: 1, 3 or 10 mg/kg q3w. More than 50% of patients were heavily pretreated, receiving at least three lines of therapy for advanced disease. ORR was 17% across doses (24% with nivolumab 3 mg/kg), with no significant difference across histology types (16.7% for squamous and 17.6% for non-squamous). Responses were durable, occurred early (50% at first assessment at 8 weeks) and were maintained after treatment discontinuation. Thirty-eight percent of responders who discontinued therapy for reasons other than PD responded for ≥30 weeks following end of therapy. Patients with PD-L1 overexpression (at least 5% of tumor membrane PD-L1 staining) seemed to have the best change in target lesion tumor burden. However, an exploratory analysis did not show any advantage in survival in the group of patients who overexpressed PD-L1 as compared with patients who did not. The 3 mg/kg q3w nivolumab dose obtained a median OS of 14.9 months, with 1- and 2-year OS rates of 56% and 45%, respectively, with no difference across histology.

Interim results of a first-line nivolumab monotherapy study in advanced NSCLC were also presented at ASCO 2014 [25]. Patients with chemotherapy-naïve NSCLC reported a 30% ORR, with two patients achieving a confirmed complete response (CR) and another one unconfirmed. More than 2/3 of responses were ongoing at the time of analysis. Tumor PD-L1 expression status seems to be related with response: ORR was 50% in PD-L1-positive patients, with no responses reported in PD-L1-negative patients. Nivolumab monotherapy had a tolerable safety profile, with a low frequency of grade 3/4 treatment-related AEs and no treatment-related deaths.

PD-1 and CTLA-4 are non-overlapping immune checkpoints in T-cell differentiation and have demonstrated antitumor synergy in murine models and melanoma patients. A combination phase I study of nivolumab and ipilimumab in patients with previously untreated advanced NSCLC is ongoing, and preliminary results (n = 49) were recently presented [36]. Two different doses of nivolumab and ipilimumab (1 mg/kg and 3 mg/kg q3w) were combined in this trial, and subsequent nivolumab-only maintenance (3 mg/kg q3w) was offered until disease progression or toxicity. Nivolumab in combination with ipilimumab provided durable ORR regardless of histology. Activity was shown irrespective of tumor PD-L1 status, suggesting that the combination may be suitable for both PD-L1-negative and PD-L1-positive patients. There was no clear relationship between efficacy outcomes (ORR, PFS and OS) and PD-L1 status, as previously reported in patients with melanoma.

Nivolumab in combination with standard platinum-based chemotherapy as front-line treatment in advanced NSCLC has also been explored in a phase I trial [37]. Preliminary results showed that antitumor activity of nivolumab added to chemotherapy was similar to that previously reported for standard platinum-based doublet regimens. ORR ranged from 33–47% across treatment arms, with OS and PFS data consistent with those previously reported in patients treated with chemotherapy alone. These results also revealed a safety profile reflecting additive toxicities of nivolumab and chemotherapy, although with no higher frequency of severe grade AEs. In patients with EGFR-mutant non-squamous NSCLC, the combination of nivolumab plus erlotinib has shown an encouraging and durable response rate, with a manageable safety profile [38].

Vaccines are potentially effective in patients with lung cancer. Maximal benefit could be attained in patients with minimal disease, such as after resection, definitive chemo-radiation and PR or CR on first-line combination therapy. Some interesting results have been obtained in NSCLC with tumor cell-derived multiple and specific antigen vaccines. Belagenpumatucel-L (Lucanix) is an allogeneic tumor cell vaccine made with four irradiated NSCLC cell lines and modified with transforming growth factor-β2 (TGF-β2) antisense plasmid, which improves the immune response. In a phase III, placebo-controlled trial in patients with stage III/IV NSCLC and disease control after first-line chemo-radiotherapy (CT-RT), Lucanix was administered as monthly intradermal injections for 18 months, followed by two injections on a quarterly basis [39]. This trial did not meet its primary endpoint (OS), but a significantly prolonged survival was shown in patients who began the vaccine within 12 weeks from the completion of front-line treatment, both in squamous and non-squamous histology.

Melanoma-associated antigen A3 (MAGE-A3) is a tumor specific antigen that is aberrantly expressed in approximately 35% of NSCLC and is not expressed on non-malignant cells, except for testicular germ cells and placental trophoblasts. MAGE-A3 vaccine is composed of the MAGE-A3 protein plus an adjuvant AS15. In a phase II randomized study, patients with resected NSCLC were randomly assigned to either MAGE-A3 (n = 122) or placebo (n = 60) [40]. After a median post-resection period of 44 months, recurrence was observed in 35% of patients in the MAGE-A3 arm and 43% in the placebo arm. No statistically significant improvement in disease-free interval (DFI)(HR 0.75, p = 0.254), disease-free survival (DFS) (HR, 0.76; p = 0.248) or OS (HR, 0.81; p = 0.454) was observed. A similar trend for DFI and DFS was revealed after a longer follow-up period (70 months). Moreover, a large randomized phase III trial with adjuvant MAGE-A3 vaccine after adjuvant chemotherapy in patients with resected stage IB through IIIA MAGE-A3 positive NSCLC failed to meet its primary endpoint [41].

Liposomal BLP-25 (L-BLP-25, Tecemotide) is a peptide-based vaccine targeting the exposed core peptide of membrane-associated glycoprotein (MUC-1), normally expressed on epithelial cells. Tumor-associated MUC-1, which is aberrantly glycosylated, is antigenically distinct from normal MUC-1 and it is associated with oncogenesis and resistance to chemotherapy. MUC-1 is overexpressed in approximately 60% of lung cancers. In a randomized phase IIB study, patients with stable or responding stage IIIB or IV NSCLC after first-line chemotherapy received L-BLP-25 plus BSC or BSC alone [42]. Patients in the vaccine arm received a single intravenous dose of cyclophosphamide 300 mg/m^2^ followed by eight weekly subcutaneous immunizations with L-BLP-25 (1000 μg). Subsequent immunizations were administered at 6-week intervals. The median survival time was 4.4 months longer (non-statistically significant) for patients assigned to the L-BLP-25 arm (n = 88) as compared to patients in the BSC arm (n = 83), with an adjusted hazard ratio of 0.739. The greatest effect was observed in stage IIIB patients, where the median survival time for the vaccine arm has not yet been reached compared with 13.3 months for the BSC arm.

A subsequent randomized phase III trial with tecemotide in patients with stage III NSCLC who received concurrent or sequential CT-RT reported no significant difference in OS with tecemotide compared with placebo in the intention-to-treat population [43]. In the subgroup of patients who received concurrent CT-RT, median OS for patients assigned to the experimental arm was 30.8 months (95% CI 25 · 6–36 · 8) as compared with 20.6 months (17 · 4–23 · 9) for those who received placebo (adjusted HR 0 · 78, 0 · 64–0 · 95; p = 0 · 016). Possible reasons for improved results with concurrent CT-RT could be the better performance status and the smaller tumor size in patients who received this treatment. Some authors recently postulated that the favorable outcome of concurrent CT-RT in different solid tumors might be explained by immunogenic cell death [44]. The activity of other promising vaccines in NSCLC, such as MUC-1 antigen-derived TG 4010 and EGF-derived CIMAvax, has been shown in phase II trials [45,46]. Survival results from phase III trials are awaited, as well as further investigations to identify patient subgroups that might benefit from these strategies.

#### Colorectal cancer

Although it is known that tumor-specific T-cells can be isolated from patients with gastrointestinal (GI) tumors, the potential use of immunotherapy to treat advanced GI malignancies is far from realization. Infiltration of T-cells into GI tumors correlates with improved prognosis, while the presence of negative regulatory factors that inhibit antitumor T-cell responses correlates with a poor prognosis [47].

PD-L1 expression seems to correlate with decreased immune activation and poor clinical outcome in GI tumors. Expression of PD-L1 in colorectal cancer occurs in about 60% of patients [48]. However, a phase I study of nivolumab for advanced or recurrent colorectal cancer reported limited activity [17].

Patients with high levels of microsatellite instability could potentially have greater benefit from immunotherapies in metastatic colorectal cancer. In this group of patients, a clinical phase I/II trial exploring the feasibility of nivolumab plus ipilimumab combination is ongoing (http://clinicaltrials.gov/ct2/show/NCT02060188). Apart from moAbs, other promising immune-therapy strategies are emerging in colorectal cancer, such as vaccination and adoptive cell therapies.

#### Gastrointestinal (non-colorectal) cancer

Monotherapy with ipilimumab was shown to be ineffective in patients with advanced pancreatic cancer unsuitable for surgery in a single-arm phase II trial that enrolled 27 patients (20 with stage IV disease) [49]. Symptoms related to disease progression limited the number of doses received by patients, with only 12 receiving at least one course. There were no responders by RECIST criteria, with only one patient who experienced a delayed response. Although ipilimumab was ineffective, the significant delayed response in one patient suggests that immunotherapeutic approaches deserves further exploration in this field.

A randomized, open-label phase II trial comparing ipilimumab versus BSC after platinum and fluoropyrimidine-based doublet first-line chemotherapy in unresectable or locally advanced/metastatic gastric cancer is ongoing (http://www.clinicaltrials.gov/ct2/show/NCT01585987). Primary endpoint is to achieve an irPFS benefit from ipilimumab as compared to BSC in patients who did not progress after first-line chemotherapy.

In patients with hepatocellular carcinoma (HCC) not suitable for local treatment strategies, survival data are very poor. Sorafenib remains standard therapy in patients with an acceptable hepatic function (usually characterized as Child class A or B). This first-generation tyrosine-kinase inhibitor (TKI) showed a statistically significant advantage in time to progression (TTP) and OS compared with placebo, both in Caucasian and Asian patients [50,51]. From a biological point of view, HCC seems to be a very complex disease, involving multiple pathways which could be targeted in order to improve outcomes. Despite this, randomized phase III trials comparing targeted therapies versus sorafenib have dramatically failed in the first-line setting [52,53]. Very poor results have also been obtained with second-line therapy in two phase III trials that compared everolimus or brivanib to placebo (Table 3) [53].

**Table 3:** **Summary of phase III trial (first- and second-line) in advanced hepatocellular carcinoma**

**Treatment**	**Principal targets**	**Patients (n)**	**Median overall survival**
First-line			
Sunitinib vs SOR	VEGFR, PDGFRa/b, c-KIT, FLT3, RET	1074	8.1 vs 10 months
Cheng et al. 2013			HR 1.31 (1.13-1.52), p = 0.0019)
Brivanib vs SOR (BRISK-FL)	VEGFR, FGFR	1155	9.5 vs 9.9 months
Johnson et al. 2013			HR 1.07 (0.94-1.23), p = 0.3116
Linifanib vs SOR	VEGFR, PDGFR	1035	9.1 vs 9.8 months
Cainap et al. 2012			HR 1.046 (0.896-1.221), p = 0.1785
Erlotinib/SOR vs placebo/SOR (SEARCH)	EGFR	720	9.5 vs 8.5 months
Zhu et al. 2012			HR 0.929 (0.781-1.106), p = 0.204
Second-line			
Brivanib vs BSC (BRISK-APS)	VEGFR, FGFR	395	9.4 vs 8.2 months
Llovet et al. 2013			HR 0.89 (0.69-1.15), p = 0.3307
Everolimus vs BSC (EVOLVE-1)	mTOR	546	7.6 vs 7.3 months
Zhu et al. 2014			HR 1.05 (0.86-1.27), p = 0.675

BSC = best supportive care, HR = hazard ratio, SOR = sorafenib.

Other interesting trials are ongoing in GI non-colorectal malignancies. PD-L1 expression accounts for about 40% of patients both in esophageal/gastric and pancreatic cancers [48] and trials have been designed to evaluate the activity and toxicity of anti-CTLA-4 in combination with anti-PD-1/PD-L1 drugs in patients with GI non-colorectal tumors. CA 209–032 is a phase I/II open-label study of nivolumab alone or combined with ipilimumab in patients with advanced solid tumors including gastric and pancreatic cancer (http://www.clinicaltrials.gov/ct2/show/NCT01928394). In the experimental arm, nivolumab is administered at 1 mg/kg or 3 mg/kg q2w in combination with standard ipilimumab dose and schedule (four doses at 3 mg/kg q3w). Tremelimumab is being evaluated in an ongoing phase II trial; accrual has completed and results are awaited. Pidilizumab is an anti-PD-L1 antibody which is able to reduce T-lymphocyte apoptosis and enhance natural-killer cell activity. A phase I/II trial in patients with solid tumors is ongoing.

#### Central nervous system tumors

Glioblastoma multiforme (GBM) is the most frequent primary tumor of the central nervous system (CNS) in adults. Despite a multimodal therapeutic approach involving surgery, radiotherapy and temozolomide-based chemotherapy, median survival does not extend beyond around 15 months. Moreover, these therapies damage normal tissue and there is a need for more specific and effective treatment that is able to selectively target tumor cells without damaging normal brain tissue. CNS has to be considered as an immunologically privileged site with the blood–brain barrier (BBB) of the cerebrovascular endothelium reducing entry of immune cells and immune mediators to the CNS. Nevertheless, despite the absence of lymphatic vessels and nodes, in pathological conditions a very well organized immunological response can develop within the CNS. Bevacizumab, an anti-VEGF MoAb, has shown activity in patients with recurrent GBM and has obtained regulatory approval for clinical use in the US [54,55]. Nevertheless, bevacizumab failed to improve survival outcomes when combined with temozolomide plus RT as front-line treatment in two large clinical trials [56,57].

Anti-EGFR targeted therapy seems to be a promising approach in malignant gliomas. EGFR variant III (EGFR vIII) is one of the most frequent mutations in GBM (about 40% of total) [58]. Variant III is a deletion in the 267 position of the EGFR extracellular domain, not expressed in normal glioma tissue but only in glioblastoma cells. The most promising peptide vaccine targeting EGFR vIII is rindopepimut (CDX-110), which contains a peptide derived from the novel fusion junction amino acid sequence of EGFR vIII. Rindopepimut is able to activate humoral and cellular immunoreactivity, and has been shown to induce EGFR vIII-specific immune responses in preclinical and clinical studies [59]. A phase II, multicenter trial was conducted to assess the immunogenicity of rindopepimut and to estimate the PFS and OS of vaccinated patients with newly diagnosed EGFR vIII-positive GBM with minimal residual disease [60]. PFS was 14.7 months in the vaccine group (n = 18) and 6.3 months in the historical control group who received temozolomide (n = 17). Median OS was 26.0 months in the vaccine group and 15.0 months in the control group. Based on these promising results, a phase II study of the safety and efficacy of rindopepimut in combination with bevacizumab is ongoing (ReACT study). Patients with GBM and EGFR vIII mutation who relapse after RT plus temozolomide are randomized to receive bevacizumab plus rindopepimut or keyhole limpet hemocyanin as a control. As bevacizumab blocks VEGF and has immunosuppressive properties, the hypothesis is that the combination could enhance the immunogenic response of rindopepimut against EGFR vIII-expressing GBM cells.

#### Gynecologic cancers

The immune system plays an active role in the pathogenesis of ovarian cancer, as well as in the mechanisms of disease progression and OS. Immunotherapy in gynecological cancers could help to revert immunosuppression and lymphocyte depletion due to locoregional and systemic treatments. CD4+ T-reg cells rapidly decrease after primary tumor debulking in patients with ovarian cancer. Similar results could also be obtained in patients “chemically debulked” with neoadjuvant chemotherapy [61]. Active immunotherapy with antigen-specific peptide vaccination is one of the most promising strategies in gynecological cancers. NY-ESO-1 is one of the most immunogenic tumor antigens and is frequently expressed both in ovarian and in vulvar cancers. Immunization with peptide epitope ESO (157–170) in patients with ovarian cancer (minimal disease) enhances the production of CD4+ and CD8+ cell clones. These cells are able to recognize NY-ESO-1 expressing tumor targets and were detectable up to 12 months after immunization [62].

Abagovomab (ACA 125) is an anti-idiotypic moAb that functionally imitates the tumor antigen CA 125, overexpressed in patients with gynecological cancers. Abagovomab is able to induce a specific anti-anti-idiotypic antibody response in about 70% of patients with advanced ovarian cancers. A median survival of 23.4 months has been observed in the group of patients with an antibody response, compared with only 5 months in patients without [63]. In a randomized multicenter phase III trial, patients with stage III-IV ovarian cancer in complete remission after primary surgery and platinum- and taxane-based chemotherapy were assigned to receive maintenance therapy with abagovomab or placebo [64]. Abagovomab was safe and induced a measurable immune response. However, no significant benefit in relapse-free survival and OS was obtained compared with placebo. Comparable results have been obtained by oregovomab, another CA-125-specific murine moAb, after front-line therapy in a favorable subset of patients with ovarian cancer [65].

The multifunctional antibody catumaxomab binds both to the EpCAM tumor cell antigen and to CD3+ lymphocytes, enhancing antitumor activity by redirecting T-cells and Fcgamma receptor I/III-positive accessory cells to the tumor. A phase II dose-escalation study investigated tolerability and efficacy in patients with ovarian cancer-induced malignant ascites [66]. Treatment with catumaxomab resulted in significant and sustained reduction of ascites flow rate, with more than 90% of patients not requiring paracentesis between the last infusion and the end of study. Tumor cell monitoring also revealed a reduction of EpCAM-positive malignant cells in ascites by up to 5 log. A subsequent phase III randomized, placebo-controlled trial revealed that tumor cell numbers and peritoneal levels of VEGF decreased with catumaxomab, whereas the activation status of CD8+ and CD4+ T-cell populations increased more than two-fold after treatment [67].

Interleukins have been extensively studied as a potential treatment strategy in advanced ovarian cancer. Human-recombinant IL-12 (rhIL-12) has an anti-angiogenic effect and, when administered intraperitoneally, enhances tumor response across local delivery of other cytokines. A phase II multicenter trial has investigated the efficacy and toxicity profile of rhIL-12 in patients with advanced ovarian cancer and peritoneal carcinomatosis (residual disease <1 cm) after primary therapy [68]. Intraperitoneal IL-12 seems to have a good toxicity profile, but only achieved SD and no responses in this group of patients. Cytokine response profiles suggest either NK or T-cell mediated effects of IL-12. A pleiotropic immunologic response was induced by rhIL-12, with both anti-tumor (driven by IFN-gamma, IP-10) and pro-tumor growth effects (VEGF, IL-8).

The tumor microenvironment can modify dendritic cell function in an immunosuppressive fashion, with strong inhibition of dendritic cell activation and maturation [69]. Dendritic cell vaccines redirect T-cell immunity from immune-suppression to pro-inflammatory anti-tumor responses. Encouraging results obtained in small studies with some PRs achieved [70,71] mean that dendritic cell vaccine strategies should be assessed in larger clinical trials. Patient populations should have minimal disease at the time of vaccination, and should preferably have completed surgery and chemotherapy. The goal is to prevent disease recurrence or progression, rather than use dendritic cell vaccination as salvage therapy in women with significant tumor burden.

Promising data with anti-PD-1 therapy in patients with platinum-resistant ovarian cancer have recently been presented [72]. Patients with recurrent or refractory disease (n = 20) received nivolumab at 1 mg/kg or 3 mg/kg dose q2w on an 8-week cycle, for a maximum of 6 cycles. Standard chemotherapy was concurrently administered every 8 weeks during the study. Combination therapy was well tolerated, with few treatment-related severe AEs. A total ORR of 17% was obtained. The 3 mg/kg cohort seemed to have the most favourable outcome, with 25% RR and 63% DCR. Biomarkers predicting response or AEs are being explored in this trial.

#### Head and neck cancers

The prognosis of patients with recurrent or metastatic head and neck cancer (mHNC) is very poor with median survival ranging from 6 to 9 months depending upon patient- and disease-related factors. EGFR is strongly overexpressed in the majority (80-100%) of patients with squamous cell carcinoma of the head and neck [73]. The EGFR-directed moAbs cetuximab and panitumumab are the mainstay of treatment combined with standard chemotherapy in patients with recurrent disease. In the EXTREME study, 442 patients with mHNC were randomized to receive platinum-based chemotherapy (cisplatin or carboplatin) plus 5-fluorouracil every three weeks to a maximum of six cycles, with or without weekly cetuximab [74]. Patients in the cetuximab arm had the option to continue anti-EGFR as maintenance treatment until disease progression or unacceptable toxicity. The addition of cetuximab to chemotherapy significantly improved median OS (primary endpoint) from 7.4 to 10.1 months (HR 0.80, 95% CI, 0.64–0.99). Median PFS also increased from 3.3 to 5.6 months (HR 0.54, 95% CI 0.43–0.67), and RR improved from 20% to 36%. Treatment with cetuximab was well tolerated. A retrospective biomarker analysis of this trial revealed that there was no predictive correlation between gene copy number and cetuximab efficacy [75].

**Figure 3:**
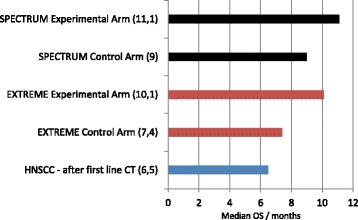
**OS improvement in the treatment of advanced mHNC adding anti-EGFR MoAbs to chemotherapy [** 74 **,** 76 **].**

The SPECTRUM trial randomized 657 patients with mHNC to receive cisplatin + 5-fluorouracil ± panitumumab [76]. The addition of panitumumab significantly improved median PFS (5.8 versus 4.6 months) but not OS (11.1 versus 9.0 months). As expected, skin toxicity was greater with the addition of panitumumab. According to the authors, P16-INK4A status could be a prognostic and predictive marker in patients who received panitumumab and chemotherapy, given that median OS was longer in the panitumumab group only in patients with P16-negative tumor status (11.7 vs 8.6 months; HR 0 · 73; p = 0 · 0115). These two trials, although not completely positive, gave some improvement in treatment outcomes in a disease with historically poor prognosis (Figure 3).

Human papillomavirus (HPV) associated with head and neck cancer generally induces a powerful immune response. Despite the development of an inflammatory microenvironment, HPV is able to persist and promote malignant transformation. The PD-1/PD-L1 immune checkpoint may play a critical role in the creation of an immunoprivileged site for viral persistence and for the subsequent development of cancer. Some authors recently published evidence of the role of the PD-1/PD-L1 pathway in HPV-positive mHNC immune resistance development [77]. They demonstrated a strong membranous expression of PD-L1 in the tonsillar crypts, the site of initial HPV infection, and the role of the PD-1/PD-L1 interaction in creating a privileged site for initial viral infection and subsequent adaptive immune resistance once tumors are established. This findings suggest a rationale for therapeutic blockade of this pathway in patients with HPV-positive mHNC. Clinical trials with nivolumab and other anti-PD1/PD-L1 drugs are ongoing.

#### Prostate cancer

Significant progress has been made in the immunotherapy of prostate cancer in recent years. From a pathogenetic perspective, prostate cancer should not be considered as a single disease. The progression from one state to another, as well as the development of castration-resistant prostate cancer (CRPC), is likely to be associated with immunological changes [78]. The development of immunotherapy strategies, both in hormone-sensitive and in castration-resistant disease, could be very promising, although defining clinical responses could be challenging in this setting of patients with no burden of disease.

In the cell-based vaccines category, sipuleucel-T is certainly the most extensively studied in prostate cancer. This vaccine is based upon autologous peripheral-blood mononuclear cells (PBMCs), activated ex vivo with a recombinant fusion protein (PA2024). PA2024 is a prostatic acid phosphatase (PAP) fused to an immune-cell activator, granulocyte-macrophage colony-stimulating factor (GM-CSF). The IMPACT trial was a double-blind, placebo-controlled phase III study that involved 512 men with asymptomatic or minimally symptomatic, castration-resistant, metastatic prostate cancer [79]. Due to a previous sipuleucel-T randomized trial that suggested a positive effect on progression in men with more differentiated disease [80], the IMPACT trial enrolled only men with a Gleason score ≤7 at diagnosis. Primary endpoint of the study was reached, with a relative reduction of 22% in the risk of death in the sipuleucel-T group compared with the placebo group. This reduction represented a 4.1 month improvement in median OS for the experimental arm. Sipuleucel-T was the first immunotherapy to reach a statistically significant survival benefit in prostate cancer. The short duration of treatment and the favorable toxicity profile were additional benefits. However, the enrolled cohort in this trial was highly selected (asymptomatic/minimally symptomatic, Gleason score ≤7) and does not necessarily reflect the patient population seen in daily clinical practice. As such, the results cannot easily be translated.

GVAX-PCa is a vaccine including a mixture of two irradiated allogeneic prostate cancer cell lines, LNCaP and PC-3, which constitutively express GM-CSF. Preclinical data suggested an additive effect for chemotherapy properly timed with G-VAX [81]. However, two randomized trials with G-VAX in combination with docetaxel in patients with CRPC have failed, both with early interruption. VITAL-1 was a randomized phase III trial with G-VAX plus docetaxel versus docetaxel alone in patients with asymptomatic metastatic CRPC which was stopped after an interim analysis showed inferiority of the experimental arm. The VITAL-2 trial had the same design, but treatment was offered to patients with symptomatic disease. The study was halted at about 1/5 of the enrolment target, due to an imbalance of deaths between the combined treatment and chemotherapy only arms (n = 67 vs 47) [82].

The role of ipilimumab in prostate cancer is not yet well established. Ipilimumab failed to meet the primary endpoint of improving OS in the randomized, phase III CA184-043 trial that included 799 patients with post-docetaxel metastatic CRPC [83]. Median OS was 11.2 months for ipilimumab vs 10 months for placebo (HR = 0.85, 95%CI = 0.72–1.00, p =0.0530). The trial met its secondary endpoint of PFS (4 months in the ipilimumab group vs 3 months in the placebo group). Pre-specified subset analysis suggested that ipilimumab improved survival in patients with more favorable prognostic factors (no visceral metastasis, lower alkaline phosphatase, and higher haemoglobin level), a finding that needs to be validated in future trials [84].

PROSTVAC is a poxviral-based vaccine targeting PSA. In a randomized phase II trial, PROSTVAC or a control vector were administered to patients with metastatic CRPC [85]. This study failed to reach its primary endpoint, with a median TTP of 3.8 months in the PROSTVAC arm and 3.7 months in the control arm. Unexpectedly, median OS greatly favoured PROSTVAC, with a difference of 8.5 months (25.1 versus 16.6 months). It has been hypothesized that this difference in results between PFS and OS, already seen in the sipuleucel-T trial [86], is due to the long-term effects of immunotherapy, which are not reflected in earlier endpoints such as TTP. Patients who died within the first 6–12 months seem to have no benefit from the vaccine. This should raise issues about evaluating outcome endpoints in trials with immunotherapy in CRPC. Immune responses may initially appear as dimensional tumoral increase, due to lymphocyte infiltration and inflammation and tumors might progress before immunotherapy has time to take effect. In randomized trials, immunotherapy should be tested earlier in patients with CRPC. Following treatments should be homogenous between the two arms, with assessment of time to subsequent progressions.

#### Bladder and kidney cancer

Although bladder cancer is often a non-muscle invasive disease at diagnosis, it shows dramatically high local and distant recurrence rates. Immunotherapy with intravesical instillation of Bacillus Calmette-Guerin (BCG) remains the most effective therapy for patients with high-risk, non-muscle invasive tumors. BCG therapy has significant limitations though, including AEs and frequent treatment failures, so new immunotherapeutic strategies are needed.

The immunomodulatory effects following brief exposure to ipilimumab in patients with urothelial carcinoma of the bladder requiring surgery have been explored in a clinical trial [87]. Treatment with ipilimumab at 3 mg/kg or 10 mg/kg was well tolerated and led to an increase of CD4+ ICOS-high effector cells. Patients were followed for a median of 20 months and 75% were free of recurrence at the time of study publication. This population of CD4+ ICOS-high cells, which was found to be increased in both tumor tissue and peripheral blood, could be a biomarker related to clinical outcome in patients with metastatic disease who received ipilimumab. A retrospective analysis in patients treated with ipilimumab for metastatic melanoma showed that a sustained increase in CD4+ ICOS-high cells is correlated with improved OS.

PD-L1 expression occurs very frequently in urothelial bladder cancer and may protect cancer cells from immune-mediated destruction by binding to its receptors PD-1 and B7.1. MPDL3280A is a human anti-PD-L1 moAb with an engineered Fc-domain that inhibits the binding of PD-L1 to PD-1 and B7.1. In a phase I study, 68 patients with bladder cancer received MPDL3280A 15 mg/kg q3w for up to 1 year. Preliminary data from this trial have been presented at ASCO 2014 [88]. More than 2/3 of patients previously received two or more regimens for advanced disease (97% one platinum-containing therapy). Treatment-related grade 3–4 AEs, mainly asthenia, occurred only in 4% of patients and there were no irAEs. Preliminary analysis (n = 20) showed a 50% ORR (43% in PD-L1-positive patients), including patients with visceral metastases at baseline. All responding patients had ongoing response at the time of clinical cut-off. Biomarker analysis revealed an increase of circulating levels of IFN-gamma, IL-18 and activated CD8+ T-cells, which represent pharmacodynamic effects. This noteworthy activity of MPDL3280A in patients with heavily pretreated urothelial bladder cancer has resulted in it being granted breakthrough therapy designation by the FDA.

Renal cell carcinoma (RCC) has long been recognized as an immunoresponsive tumor, with spontaneous regressions occurring on rare occasions. Treatment with high dose interleukin-2 (IL-2), although with significant toxicity, could lead to typically durable CRs in a small percentage of patients [89]. About 2/3 of clear cell RCC patients had PD-L1 expression, and most had TILs in primary RCC. Most patients with high PD-L1 expression on either tumors or TILs had an advanced disease stage, worse prognosis and rapid metastatic progression [90].

Patients (n = 43) with metastatic RCC (favorable/intermediate MSKCC score; 80% treated with at least one prior therapy) were randomized in a phase I study to receive nivolumab 3 mg/kg + ipilimumab 1 mg/kg (arm N3 + I1) or nivolumab 1 mg/kg + ipilimumab 3 mg/kg (arm N1 + I3) q3w for 4 doses, followed by nivolumab 3 mg/kg q2w until progression or toxicity [91]. Severe grade treatment-related AEs were most frequently reported in the N1 + I3 arm (60.9% vs 28.6% of patients), mainly GI and hepatic. No grade 3–4 pneumonitis was observed. ORR was 43% in the N3 + I1 arm and 48% in the N1 + I3 arm, with about 80% of ongoing responses at the time of data cut-off. Responses occurred at the time of first tumor assessment (week 6) in about 50% of responding patients. ORR data suggest, for the ipilimumab plus nivolumab combination, greater activity than previously reported with nivolumab or ipilimumab monotherapy in RCC [17,92].

#### Breast cancer

Breast cancer has not historically been considered as an immunogenic tumor when compared with diseases such as melanoma and RCC, which have used immunotherapy with some success. Unexpectedly, the ability to profile breast tumors on a molecular level has revealed that some breast cancers demonstrate a high level of immunoregulatory gene activation. Some immune system effectors or regulating factors have been extensively studied as prognostic factors or as predictors for good response to therapy in breast cancer. FOXP3 is a member of the forkhead/winged-helix family of transcription factors involved in regulating immune system development and function. This gene plays a crucial role in the generation of CD4+ CD25+ T-regs. The loss of FOXP3 function leads to a lack of T-regs, resulting in lethal autoaggressive lymphoproliferation, whereas overexpression of FOXP3 results in severe immunodeficiency [93]. High levels of T-regs have been reported in peripheral blood, lymph node, tumor specimens, and ascites of patients with different solid tumors, including breast cancer. The intratumoral expression of FOXP3 may be an indicator that tumor-infiltrating T-regs cells influence antitumor immunity: for this reason its expression might be a potential prognostic marker [94]. The expression patterns of FOXP3 were examined by immunohistochemistry in primary breast cancer specimens from patients enrolled in the Milan 1 and Milan 3 trials [95]. FOXP3 expression in tumors was associated with worse OS probability and the risk increased with increasing FOXP3 immunostaining intensity. FOXP3 was also a strong prognostic factor for distant metastases-free survival but not for local recurrence risk. In multivariate analysis, FOXP3 was an independent prognostic factor, as were the hazard ratio of FOXP3 expression and lymph node positivity. These data have identified FOXP3 expression as a new independent prognostic factor in breast carcinoma, which might help to improve the selection of patients for appropriate therapy.

Many of the current treatments in breast cancer have an immunogenic effect within the tumor microenvironment. For example, the neoadjuvant administration of taxanes in locally advanced disease increases the levels of TILs within the tumor. In the metastatic setting, docetaxel increases levels of Th1-associated cytokines (IL-2, IFN-γ) while decreasing negative inflammatory markers such as tumor-necrosis factor beta (TNF-β).

In the setting of breast cancer, PD-1-positive T-lymphocytes have been associated with high histological grade, ER-negative status, and intense lymphocytic infiltration [96]. PD-L1 positivity in breast cancer epithelial cells and TILs has been associated with similar negative prognostic factors [97]. Interestingly, a study in an immunocompetent mouse model of HER2-positive breast cancer provided evidence for a therapeutic synergy between trastuzumab and anti-PD1 MoAb, pointing towards a promising drug combination [98].

## Conclusions

There is an ongoing need for new treatment modalities in patients with advanced solid tumors. Surgery, radiation and cytotoxic/targeted therapies are currently the mainstay of treatment, but the mortality rate remains high for most patients with advanced or metastatic disease. Immuno-oncology is a medical area that focuses on the development and delivery of new therapies to generate an effective immune response against cancer. Improving human immune system responses has long been thought as a promising approach against solid tumors, although with conflicting results.

In the last five years, the approval of sipuleucel-T for patients with prostate cancer and ipilimumab for patients with previously-treated unresectable or metastatic melanoma has renewed great interest in this field, with very promising initial results in other solid tumors. These agents target the immune system, so they have the potential to offer a durable cancer control across a variety of tumor types, including those that were not historically considered likely to respond to immune manipulations. Follow-up from phase II and III trials consistently show a plateau in survival curves for patients treated with ipilimumab (about 20%) that is maintained for an extended period, as evidenced by follow-up of up to 10 years [99]. Immunotherapies generally have a well-tolerated toxicity profile, with limited long-term damage upon normal tissues. Ongoing research in this field will help us to address unmet needs and to understand how immune-oncology may advance current standards of care and eventually improve survival outcomes.
